# Ectopic thyroid tissue: unusual differential diagnosis of cervical paraganglioma

**DOI:** 10.11604/pamj.2017.27.43.11495

**Published:** 2017-05-16

**Authors:** Houda Chahed, Ghada Kharrat, Rim Bechraoui, Jihene Marrakchi, Azza Mediouni, Mohamed Ben Amor, Rim Zainine, Nejeh Beltaief, Ghazi Besbes

**Affiliations:** 1 Department of Oto-Rhino-Laryngology and Cervico-Facial Surgery of ’Rabta’ Hospital Tunisia, Medecine Faculty, El ManarUniversity, Tunisia

**Keywords:** Ectopic thyroid, MRI, paraganglioma, surgery

## Abstract

Ectopic thyroid tissue (ETT) lateral to the midline is rare. Its occurrence in the carotid bifurcation is exceptional. We present a 45 years woman who consulted with a slow growing right cervical swelling. Clinical examination Ultrasonography, contrast enhanced CT and cervical MRI concluded to a paraganglioma. Intra-operatively, the tumor didn’t have the characteristic aspect of a paraganglioma. Complete excision was performed. Histology concluded to an ectopic micro-vesicular thyroid adenoma.Previous literature was reviewed to summarize clinical and radiologic characteristics of such rare entity. Despite its rarity, ETT must be included in the differential diagnosis of cervical paraganglioma.

## Introduction

An ectopic thyroid gland is an uncommon embryological anomaly, defined as thyroid tissue not located anterolaterally to the second to fourth tracheal cartilages [1]. Lingual site is the most common ectopic location accounting for 70-90% of cases [2]. However, ectopic thyroid tissue lateral to the midline is rare. It's occurrence in the carotid bifurcation is exceptionaland usually misdiagnosed as a paraganglioma [3, 4]. We present the first patient from North Africa with laterally located ectopic thyroid tissue (ETT) mimicking a carotid body tumor in association with normal thyroid gland. Previous literature was reviewed to summarize the clinical and radiologic characteristics of such rare entity. Despite its rarity, lateral ETT must be included in the differential diagnosis of cervical paraganglioma.

## Patient and observation

**Figure 1:**
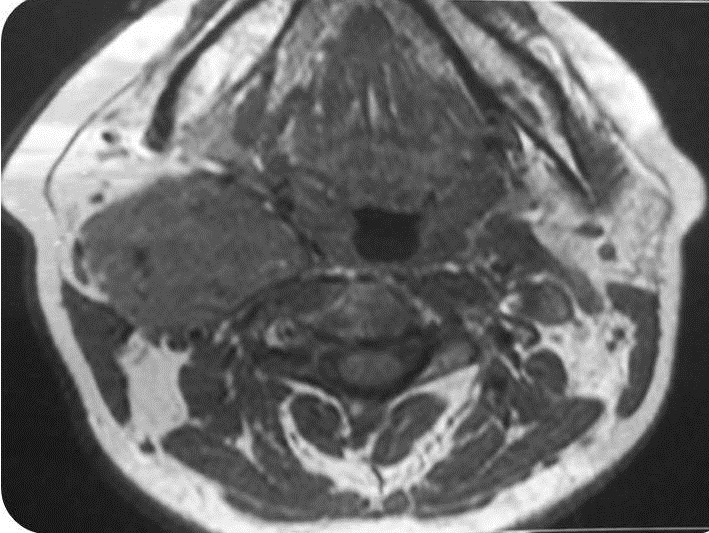
Cervical MRI; limited mass, iso intense in T1 sequence

**Figure 2:**
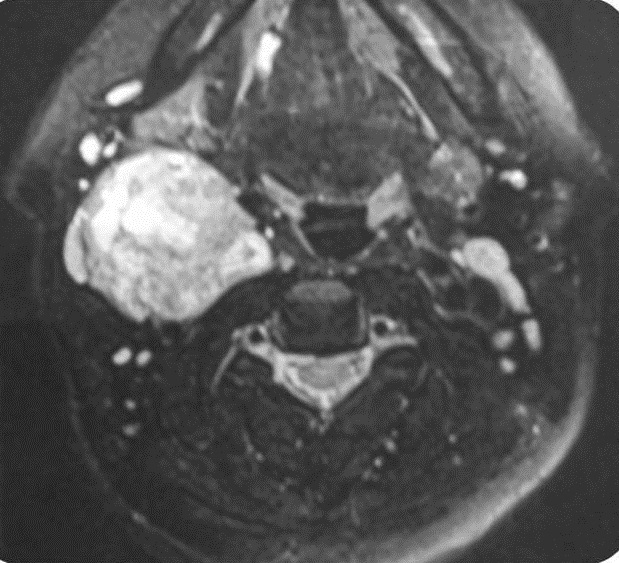
Hyperintense with heterogeneous appearance (pepper and salt) in sequence T2 located in the right carotid space

**Figure 3:**
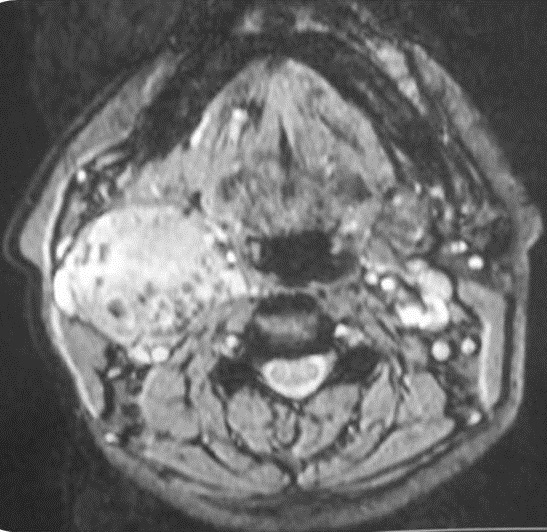
Intense enhancement after Gadolinium injection measuring 40∗49∗52 mm

A 45 years woman consulted with an isolate slow growing right latero cervical swelling evolving over 4years. Clinical examination revealed a 5cmpounding, firm and well limited tumefaction. It was mobile in the horizontal direction but not at the cranio-caudal one. The rest of the clinical examination was insignificant. Ultrasonography revealed a well limited heterogeneous, hyper vascularized tumor measuring 58*42 mm.Thyroid gland was in normal place with a right lobe node measuring 3.4 mm. Cervical MRI Figure 1, Figure 2, Figure 3 concluded to a right carotid space tumor, measuring 40*49*52 mm,which was in hypo signal T1, hyper signal associated to a salt and pepper appearance on T2 sequences with an intense enhancement following Gadolinium. The diagnosis of a carotid body paraganglioma was retained. Intra-operatively, the tumor did not have the characteristic macroscopic aspect of a carotid body tumor. It englobates partially the primitive carotid artery but was very easily cleavable of the carotid. Complete excision was performed under general anesthesia. Histologic examination concluded to an ectopic micro-vesicular thyroid adenoma. The patient was symptom free and euthyroid over the next year without evidence of recurrence.

## Discussion

Laterocervical ectopic thyroid tissue is a very rare condition. Approximately 1 to 3% of all ectopic thyroids are located in the lateral neck [5]. It has been usually located in the submandibular region. Its occurrence in the carotid bifurcation is exceptional and to our knowledge only 4 cases have been reported in the literature [1, 3, 4, 6]. The ETT is the only functional thyroid tissue in 70% to 100% of cases. Consequently, the simultaneous finding of lateral neck ETT and a functional thyroid gland in its normal location is extremely rare [7]. Etiopathogeny remains unclear. Laterally located ETT could be explained either by thecell rest left during the migration of the thyroid gland or by the maldescent of lateral anlarge of the gland during its development. Another explanation for the mechanism of ETT is detachment of evaginated thyroidtissue from lateral lobes of thyroid primordia that remain lateralized in the neck [8]. Ultimobranchial body origin may be another origin of such ectopic thyroid tissue. In all cases of ETT in the carotid space the lesion was located in the right side. [1, 3, 4, 6]. Such finding may have an embryologic explanation or be just a coincidence. Clinically, most of patients with laterally located ETT are asymptomatic. Hypothyroidism was found in 33% of patients with ectopic thyroid and it was higher in patients without normal placed thyroid gland [2].

Cervical ultrasonography, CT, and MRI are good methods for characterizing the lesion. Usually differentiation with carotid body paraganglima is not obvious even with arteriography [4]. In the four cases reported in the literature imaging exploration evocated a paraganglioma in three cases [3, 4, 6]. In one case, the diagnosis of paraganglioma was ruled out face to lesion situated anteromedially to the external carotid artery on CT Scann without vascular flow voids on MRI. However, in that case, fine needle aspiration concluded to a pleomorphic adenoma rather than a thyroid origin [1]. Nevertheless, Ultrasound-guided fine needle aspiration for extra thyroidal neck masses remains contributive to diagnosis when it is practiced by experienced hands [9]. Concerning carotid space located ETT. It is an exceptional entity [1, 3, 4]. The cases reported in the literature are summarized in table n°1. The diagnosis of ETT is difficult in spite of modern techniques of evaluation such as cervical CT scan and MRI. Diagnosis was then made post operatively on histopathologic examination. The rate of malignancy seems to be higher than in normally located thyroid with about 80% of ETT malignancies are papillary carcinoma [10].

## Conclusion

Even it is exceptional, ETT should also be taken into consideration in the differential diagnosis of lateral neck masses especially if this one is located at right side. This is even important because these masses could be the only functioning thyroid tissue. CT scan, MRI, I^123^thyroid scintigraphy and FNAC are valuable diagnostic methods that provide high sensitivity and specificity when they are combined.

## Competing interests

The author declare no competing interests.
